# Evaluating the individual, situational, and technological drivers for creative ideas generation in virtual communities: A systematic literature review

**DOI:** 10.3389/fpsyg.2022.978856

**Published:** 2022-09-12

**Authors:** Xin Zhao, Chunzhen Wang, Jianzhong Hong

**Affiliations:** ^1^Information School, University of Sheffield, Sheffield, United Kingdom; ^2^Key Laboratory of Adolescent Cyberpsychology and Behavior (Ministry of Education), School of Psychology, Central China Normal University, Wuhan, China

**Keywords:** online creative ideas generation, virtual communities, contributing factors, systematic literature review, online collaboration

## Abstract

The setting in which people generate ideas and work collaboratively to solve problems is gradually shifting from traditional face-to-face communities to virtual communities. Virtual communities are, therefore, becoming a new source of creative ideas. Nevertheless, online creativity is not without challenges. The main obstacle seems to be a lack of active engagement from participants within these virtual communities, resulting in a low quality and quantity of creative content when compared to traditional methods of creation. Research suggests that successfully generating creative ideas online involves sustained, active engagement among collaborators. A number of studies have investigated various factors related to the generation of creative ideas within virtual communities. However, a comprehensive understanding of contributing factors remains elusive. This study examines past research on the factors that drive creative ideas generation in online creative communities through a systematic literature review. The study seeks to analyze research findings over the past decade and provide an overview of the main driving factors, research areas, research trends, and implications for future research. Web of Science and Scopus were used to identify relevant articles, while Google Scholar was used to minimize the risk of missing any valuable data related to the aim of this study. The results provide an overview of the studies examining creative ideas generation within virtual communities. By approaching the subject matter from three primary perspectives (individual, situational, and technological), this paper identifies influencing factors associated with the successful generation of creative ideas online. The results of the paper also provide an overview of the research methods and guiding theories adopted by current researchers. The paper concludes with research trends and recommendations for future research.

## Introduction

The setting in which people generate ideas and work collaboratively to solve problems is gradually shifting from traditional face-to-face communities to online communities (Bugshan, 2015; Lee et al., 2019). Due to the flourishing of the Internet, people have formed various types of online groups (e.g., interest-based virtual communities, relationship-based virtual communities, etc.) and utilized a wide range of technologies (e.g., social media networks, online forums, etc.) to exchange ideas. Virtual communities are, therefore, becoming a new space for creative ideas generation (Martinez, 2017; Richard et al., 2018). Nevertheless, online creativity is not without challenges. The main obstacle seems to be a lack of active engagement from participants within these virtual communities, resulting in a low quality and quantity of creative content when compared to traditional methods of creation (Shan et al., 2020). Research suggests that successful online creative communities involve sustained, active engagement among collaborating partners (Chen et al., 2017; Piyathasanan et al., 2018). A number of studies have attempted to explore various contributing factors to a successful process of generating creative ideas (Zhang et al., 2015; Hawlina et al., 2019; Nevo et al., 2020). Nevertheless, studies based on traditional face-to-face creative communities tend to focus on the individual and situational perspectives, which do not consider the technological perspectives necessary for online collaboration. Whereas studies based on virtual communities tend to adopt an information systems perspective with a focus on technologies and overlook both the individual and situational perspectives (Chen et al., 2021). There is an urgent need to develop a comprehensive understanding of contributing factors in order to enhance the creative ideas generation process in virtual communities.

### Creative ideas generation

In a variety of contexts, the ideas generation process involves putting together a number of possible solutions to solve a problem (Girotra et al., 2010), which is often referred to as “brainstorming” in the field of social psychology (Osborn, 1957) or associated with the retrieval of knowledge between long-term and working memories in the field of cognitive psychology (Mumford et al., 1991). First and foremost, creative ideas generation involves creativity, which is formally defined as the development of novel and useful ideas (Paulus and Yang, 2000). Research suggests that creative ideas generation is the prerequisite for innovation (Škerlavaj et al., 2014) and the implementation of these ideas are essential for the adaptivity and sustainability of organizations (Mitchell and Walinga, 2017; Calic et al., 2020). The traditional ideas generation process focuses on the quality of face-to-face interaction and typically involves generating large volumes of ideas, while refraining from criticism, remaining open to unusual ideas, as well as building upon and combining the ideas of various members (Osborn, 1957). However, due to widespread use of information and communication technologies, online creative ideas generation or electronic brainstorming (EBS) has emerged as a new form of ideas generation (Kay, 1995). Through online creative ideas generation, members can interact and exchange ideas through computer technologies, which allows for a level of anonymity that cannot be achieved in a face-to-face context (Kay, 1995; Gu et al., 2020). As a result of this new form of ideas generation, researchers are encouraged to examine the technologies adopted as well as the interactions between virtual members and between members and virtual platforms.

### A typology of virtual communities

In order to develop a strong understanding of creative ideas generation within virtual communities, it is necessary to look at the types of virtual communities that could facilitate members to come together and exchange ideas. An online or virtual community is defined as “groups of people with common interests and practices that communicate regularly and for some duration in an organized way over the Internet through a common location or mechanism” (Ridings et al., 2002, p. 273). Until now, many researchers have tried to categorize various types of virtual communities. Nevertheless, a consistent typology of virtual community (VC) remains lacking (Martínez-López et al., 2016). Researchers from different disciplines may categorize VCs according to a lens that is of primary importance to their own disciplines. For example, researchers of information studies often categorize VCs according to the nature of the technology being adopted, while sociologists may categorize VCs according to the nature of the social interaction taking place, and business researchers may categorize VCs according to the types of organizations involved (commercial vs. non-commercial) (Porter, 2004). Perhaps one of the most widely accepted typologies across disciplines is that of Hagel and Armstrong (1997), which groups online communities into four main categories based on individuals' basic needs. Interest-based VCs consist of a dispersed group of people who share interests and expertise on a specific topic. Relationship-based VCs consist of people with similar experiences who come together and form meaningful personal relationships through sharing experiences, such as patient-support forums. Fantasy-based VCs provide people with opportunities to explore the virtual world of fantasy and entertainment while trying out new personas (e.g., avatars). The last category of VCs are transaction-based VCs, which meet the economic transactional needs of individuals. Hagel and Armstrong developed this framework in the 90s, but transaction-based VCs have experienced significant advancement since then. In this research, we redefine transaction-based VCs as transaction-induced VCs, which promote the exchange of information and opportunities between individuals and/or organizations that can potentially lead to transactions or enhancements of products and services. Examples of transaction-induced VCs include online brand communities, crowdsourcing communities, open innovation communities, etc.

### Individual, situational, and technological contributors for creative ideas generation

Research suggests that generating creative ideas is both a psychological and social process that is highly dependent on the characteristics of individual members and the interactions between members within a community (Škerlavaj et al., 2014; Barrett et al., 2021). A number of studies have explored individual and situational factors that influence the creative ideas generation process. For example, Somech and Drach-Zahavy (2013) found that individual factors, such as the characteristics of team members and the team climate, are essential to team creativity. At an individual level, they argue that team composition is a concomitant factor in promoting creativity, as team composition involves surface-level composition variables, including members' demographic characteristics (e.g., educational level, age, etc.) and deep-level composition variables, including members' psychological characteristics (e.g., personality, values, etc.). In the same vein, Baer (2012) argues that individual characteristics, such as members' motivations, are essential for ideas generation. Whereas on a situational level, team climate (e.g., team vision, participative safety, task orientation, and support for innovation) has positive effects on overall creativity. Similarly, Škerlavaj et al. (2014) demonstrate that the role of perceived supervisor support is salient to the creativity of team members. In addition to the individual and situational perspectives of creative ideas generation, Chen et al. (2021) notice that a new focus on the technological perspective has emerged, which explores the affordance of technologies adopted as well as the interactions between members and technologies. This new perspective could arguably be due to a recent shift from traditional face-to-face creative communities toward increasingly popular online creative communities (e.g., crowdsourcing communities, idea banks, social media networks, etc.). For example, Oldham and Da Silva (2015) and Han et al. (2020) argue that textual knowledge generated from crowds on social networking sites allows members to be exposed to more unique and diverse information and, therefore, boosts the creativity generation process. Similarly, Zhu et al. (2019) suggest that online feedback from members within VCs can promote the exchange of knowledge necessary for ideas generation.

Despite the importance of creative ideas generation and the popularity of VCs, knowledge of the specific factors that influence the creative ideas generation process in VCs remains limited (Schuhmacher and Kuester, 2012; McGrath et al., 2016; Chen et al., 2021). Most studies tend to focus on the technological perspective when it comes to VC-based ideas generation or explore the individual and situational perspectives within traditional face-to-face contexts. There is an urgent need to fill the gap and develop a comprehensive understanding of the contributing factors of creative ideas generation in VCs. This paper examines past research on the factors that drive creative ideas generation in VCs through a systematic literature review, while seeking to analyze empirical findings over the past decade to provide an overview of the main driving factors, research areas, research trends, and implications for future research. Our study was guided by the following research questions:

- How and in which contexts has creative ideas generation been investigated within virtual communities?
- How are creative ideas generation studies conducted within various types of virtual communities?
- Which contributing factors have been explored by researchers?
- Which guiding theories have been adopted regarding the contributing factors?

## Materials and methods

This paper was guided by the PRISMA (Preferred Reporting Items for Systematic Reviews and Meta-Analysis) checklist (Page et al., 2021). This updated guideline provides methodological advice on our approach to identifying, selecting, synthesizing and reporting studies to address our research questions. In this section, we outline the design and implementation of this systematic review, including our search strategy, data sources, study selections, and issues of quality and bias.

### Databases and search strategies

Web of Science and Scopus were used for identifying relevant articles, while Google Scholar was used to minimize the risk of missing any valuable data related to the aim of this study. The search process was supported by the library service of the University of Sheffield (UoS). Two consultation sessions with UoS librarians were conducted to finalize the key search terms. The following search terms were used to find articles: participant engagement/intention/commitment/motivation, online/virtual creative communities, online/virtual creative space, online/virtual creative collaboration, online/virtual creative ideas generation, online/virtual creative clusters, virtual creative space, and online innovation communities. Boolean searches (AND, OR, and NOT) were used to obtain proper search results. The recommendation systems of the online databases were used to locate similar articles (e.g., “people who viewed this article have also viewed the following articles”, “articles of a similar topic”, etc.) Only English articles were included in the search.

### First search and screening

The initial search returned 601 articles in Excel, including those from Web of Science (*n* = 327) and Scopus (*n* = 274). Out of all the articles identified, only two were duplicates and, thus, removed. After the first screening, only peer-reviewed publications written in English and published within the past decade were included (*n* = 509). Therefore, the criteria for the first screening were:

- Non-duplicated studies
- Studies published within the past decade
- Studies produced in English
- Peer-reviewed journal articles

### Second screening

Prior to the final selection, all abstracts of the remaining articles (*n* = 509) were screened by the research team. Articles that did not address the motivation/driving factors of online creative communities were excluded. Books and book chapters were excluded as they often provided conceptual discussions or practical guides rather than reporting new research findings. Conference papers were excluded as they may undergo a less rigorous review process than journal papers. The number of selected articles after the second screening was 116, including Web of Science (*n* = 68) and Scopus (*n* = 48).

### Retained papers

The research team (three researchers) downloaded and read each of the 116 papers retained after the second screening in their entirety. A final agreement was reached among the research team to include 68 papers that fulfilled the following criteria:

- addressing the motivation/driving factors of online creative communities
- online collaboration only

**Figure 1:**
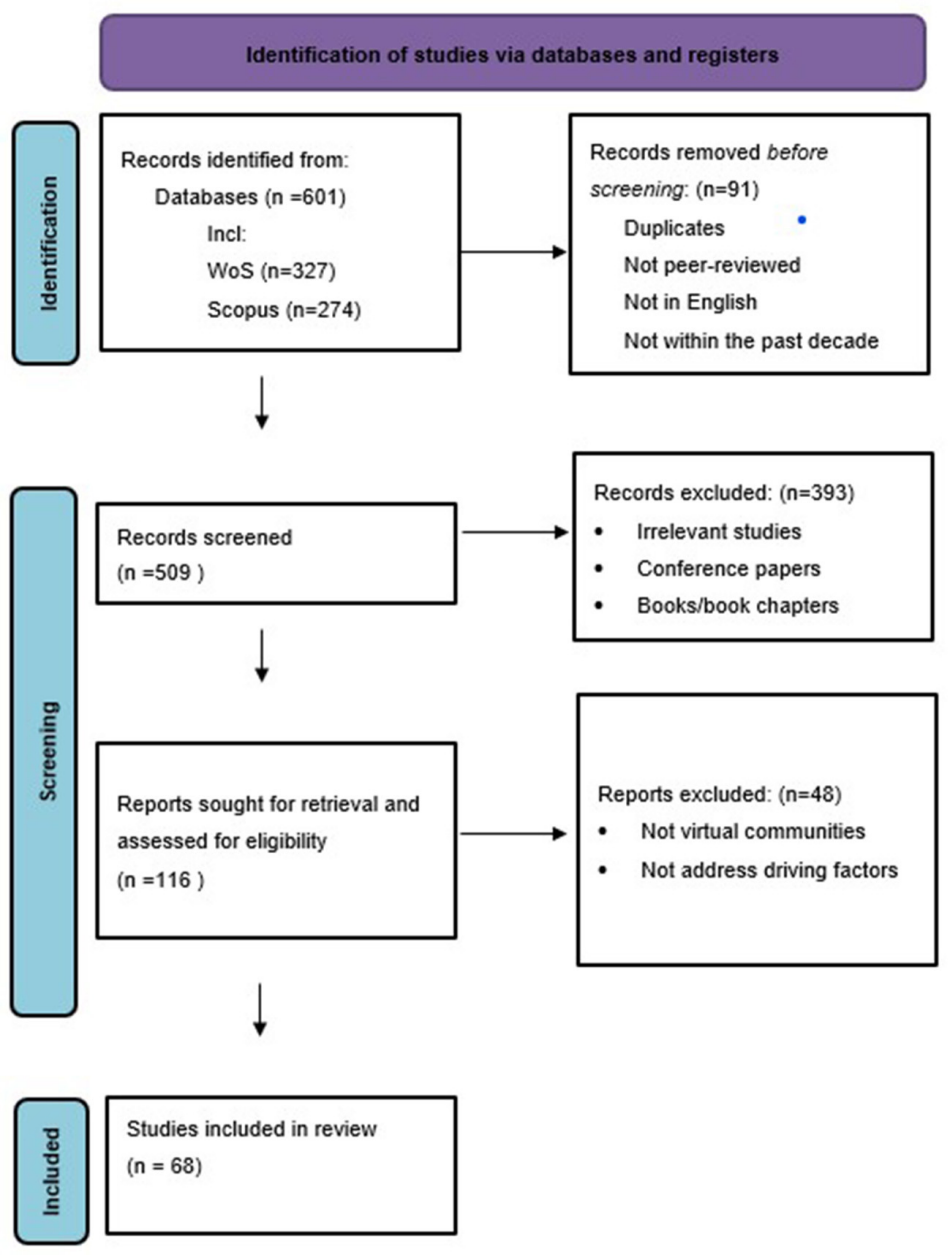
PRISMA flow diagram.

The included studies were thoroughly reviewed by each researcher and cross-examined twice by other researchers within the team to reduce potential bias. A discussion meeting was held after each round of screening to resolve disagreements and achieve consensus between researchers. The design of the review and selection of papers can be found in Figure 1.

## Results

### How and in what contexts has creative ideas generation been investigated within virtual communities?

**Figure 2:**
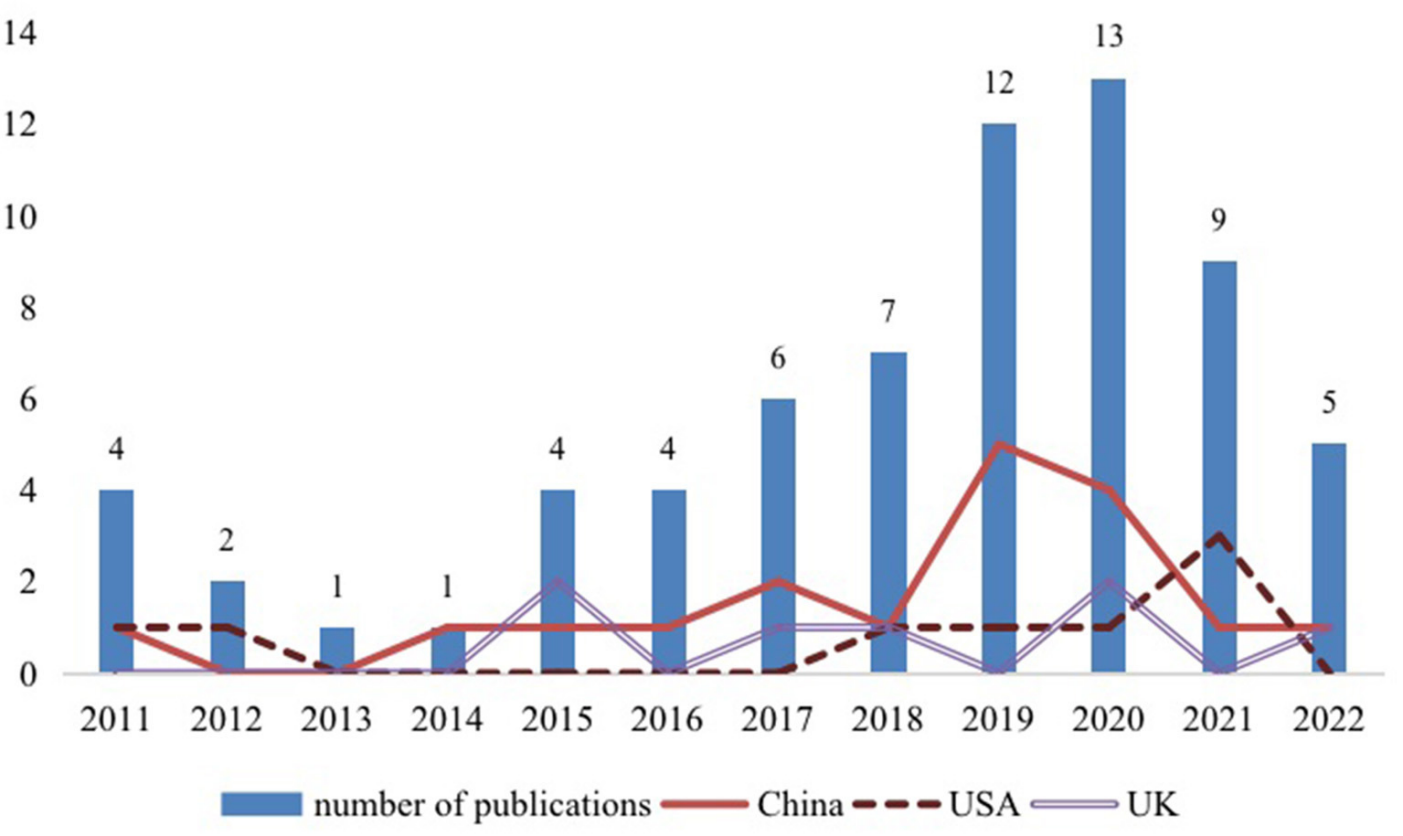
Selected articles by year and countries.

Figure 2 illustrates the number of publications per year and by country from 2011 to 2022. Among all of the selected 68 articles, a large number were published in the last 6 years, especially in 2019 and 2020, indicating an increase in interest among researchers for creative ideas generation in VCs. With regard to the publications' geographical distribution, China carried out the most studies, with 18 articles published (five of which were published in 2019). A further eight studies were carried out in the USA, while seven studies were carried out in the UK. Some of the remaining studies were carried out in other countries [e.g., Australia (*n* = 4), Italy (*n* = 4), Austria (*n* = 4), India (*n* = 3), Belgium (*n* = 3), Malaysia (*n* = 2), France (*n* = 2), Netherlands (*n* = 2)].

**Figure 3:**
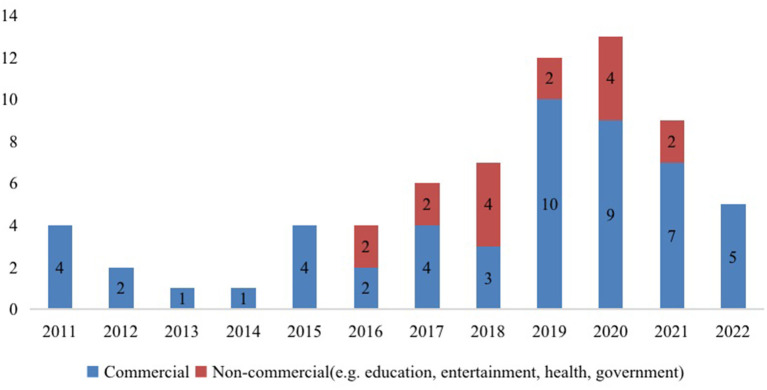
Selected articles by setting (commercial vs. non-commercial).

Regarding the setting of online creative ideas generation, the articles were classified into the following types: commercial and non-commercial. Figure 3 shows that research is primarily published in commercial contexts (*n* = 52). From 2011 to 2022, research was published yearly in commercial contexts, with the most being published in the last 4 years. Since the beginning of 2016, 16 studies have been conducted in non-commercial settings (e.g., education, entertainment, health, and government), suggesting that creative ideas generation is increasingly popular in such settings.

### How are creative ideas generation studies conducted within various types of virtual communities?

**Figure 4:**
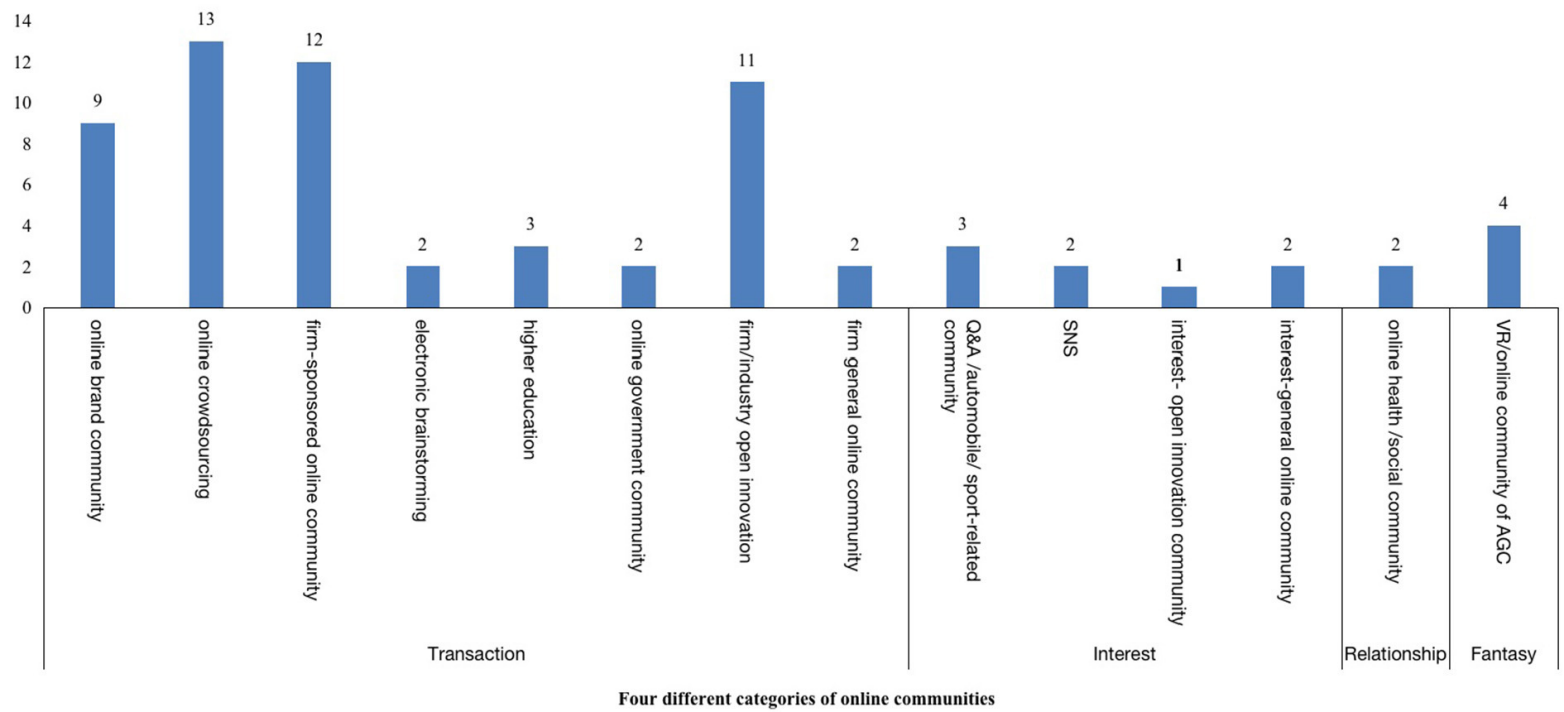
Selected articles by types of virtual communities.

Hagel and Armstrong (1997) categorized VCs into four types according to the basic needs of community members, namely: transactions, interests, relationships, and fantasies. As can be seen from Figure 4, online creative ideas generation is primarily conducted in transaction-induced communities (*n* = 54), including online brand/government communities, online crowdsourcing communities/contests, firm-sponsored online communities, and electronic brainstorming communities. Most articles within this VC category were published in the context of online crowdsourcing (*n* = 12) and firm-sponsored online communities (*n* = 13), followed by firm/product open innovation (*n* = 11) and online brand communities (*n* = 9). The interest-based VC also received considerable attention from researchers (*n* = 8), including Q&A/automobile/sports communities, social networking sites (e.g., fashion), and professional open innovation platforms. Compared to transaction-induced and interest-based VCs, less attention has been received by fantasy-based VCs (*n* = 4), such as virtual game communities and relationship-based VCs (*n* = 2) like online health forums and support communities.

**Figure 5:**
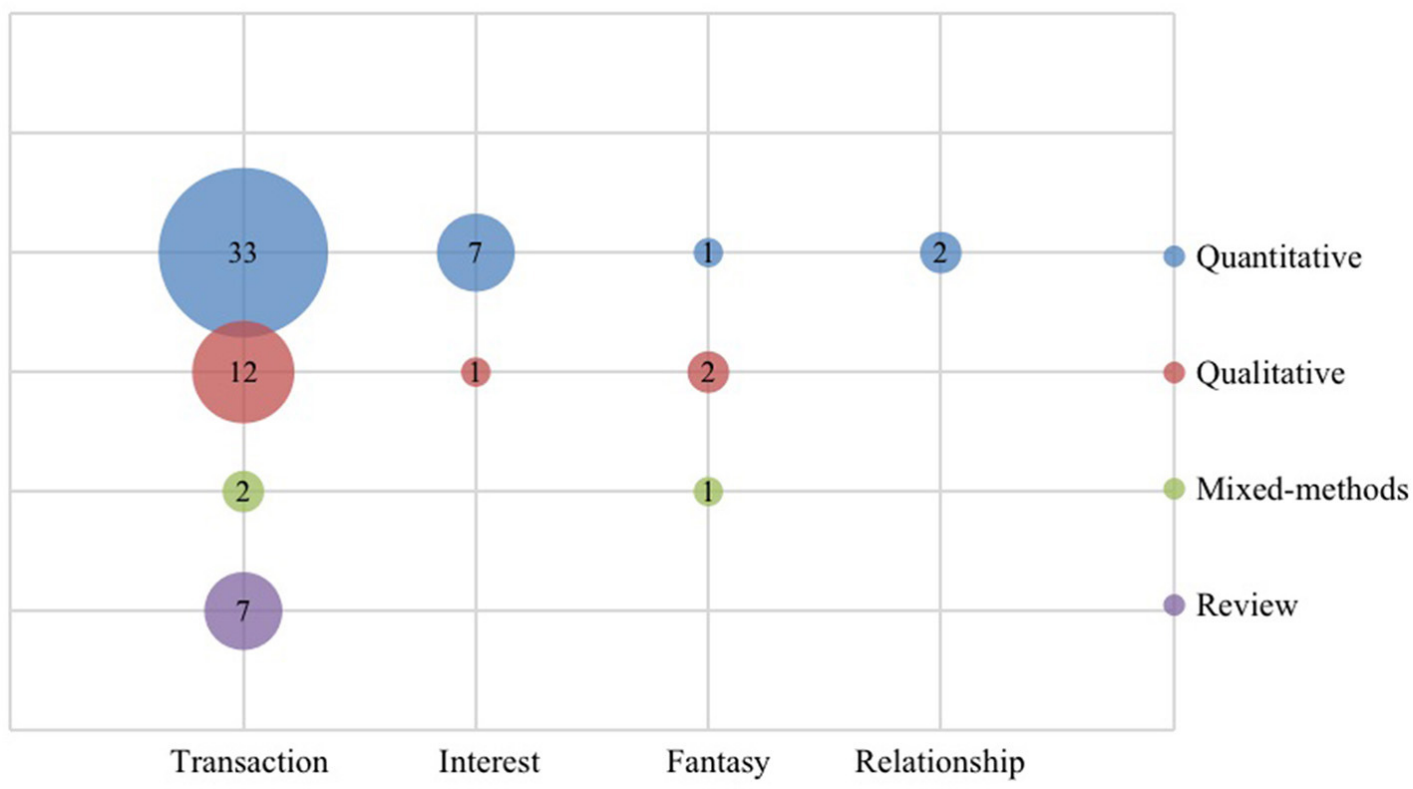
Selected articles by methods and types of virtual communities.

The research design of the selected articles includes quantitative, qualitative, mixed-methods, and review studies. The results indicate that the number of quantitative studies was most extensive (*n* = 43), followed by qualitative studies (*n* = 15) and reviews (*n* = 7); while only 3 studies were conducted using a mixed-methods approach. Although combining quantitative and qualitative methods is a strong aspect of empirical research, we can see that mixed methods have not received enough attention in online creative ideas generation. Figure 5 presents the number of publications per research method combined with four community contexts. In the transaction-induced VC context, most of the studies applied a quantitative method (*n* = 33), followed a qualitative method (*n* = 12). All reviews (*n* = 7) were carried out in this context. In the context of interest and relationship-based VCs, most studies are quantitative. For fantasy-based VCs, qualitative methods were used in two studies, followed by a quantitative study and a mixed-methods study.

### Factors influencing creative ideas generation in virtual communities: Individual, situational, and technological perspectives

**Figure 6:**
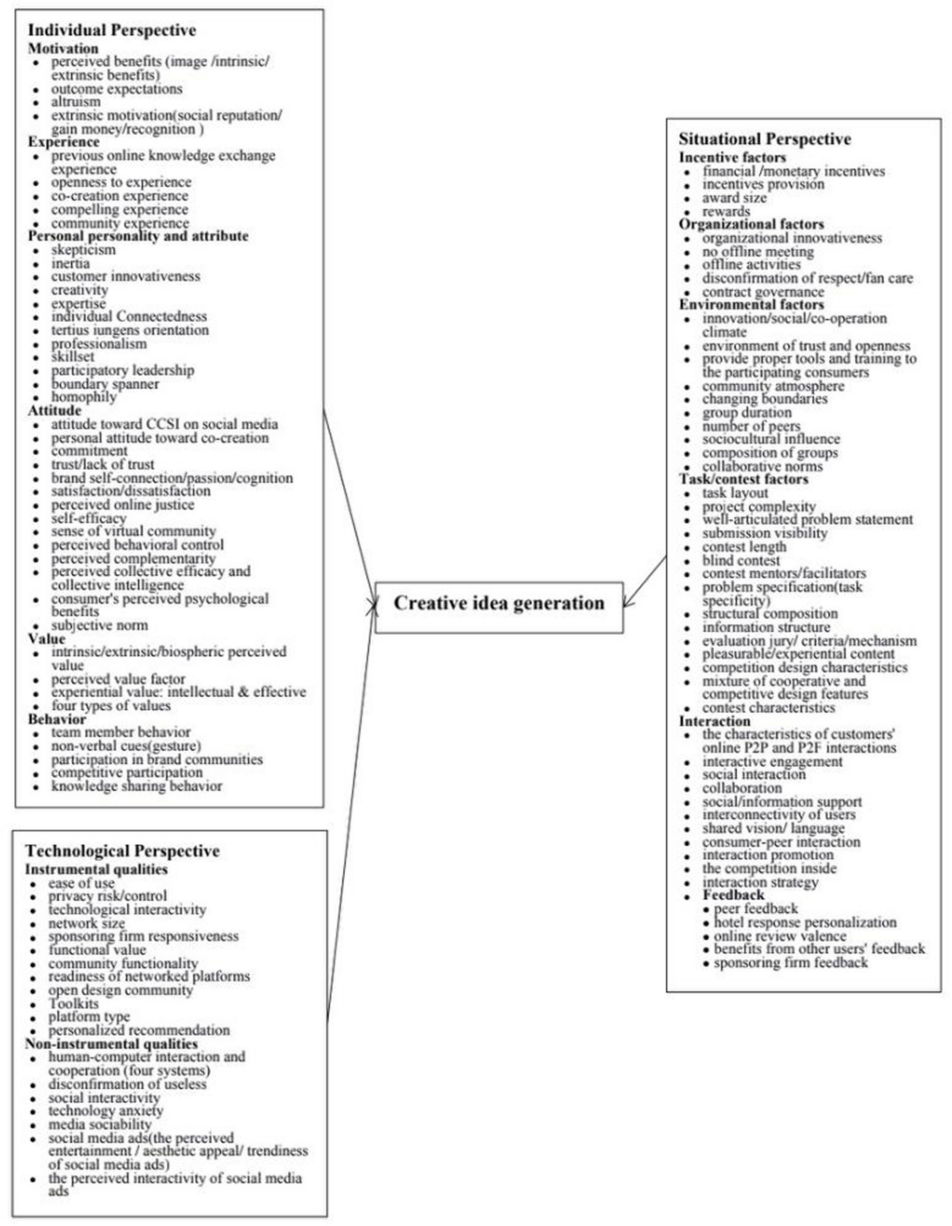
Influencing factors of online creative ideas generation.

Data analysis shows three main perspectives for creative ideas generation within VCs: individual, situational and technological. For each of these perspectives, there are various subthemes (i.e., driving factors), which are listed in Figure 6.

#### An individual perspective

An individual perspective looks at the characteristics of individual members (Rashid et al., 2019; Priharsari and Abedin, 2021) that contribute to the creative ideas generation within VCs. According to the data, the individual perspective is associated with six subthemes/factors: individual motivation, previous experience, personal personality and attribute factors, attitude, value and individual behavior. Our data suggests that research adopting an individual perspective of online creative ideas generation tends to focus on these six subthemes (see Figure 6). For example, some studies examined the intrinsic and extrinsic motivation of individual members (Zheng et al., 2011; Bettiga et al., 2018; Al-kumaim et al., 2021), while others focused on the members' behaviors (verbal and non-verbal cues) (Seeber et al., 2013; Liao and Wang, 2019).

#### A situational perspective

A situational perspective that examines creative ideas generation within VCs is often defined as a team climate (Somech and Drach-Zahavy, 2013), which describes the overall environment that enables team members to interact with tasks and each other within VCs (Füller et al., 2019; Rashid et al., 2019; Marmat, 2021). Our data suggests that research adopting a situational perspective of online creative ideas generation tends to focus on the following six subthemes: incentives, organizational factors, environmental factors, tasks/contests, interactions (among members as well as between members and organizations), and online feedback. For example, some studies examine how monetary incentives could be used to boost the generation of creative ideas online (Chen et al., 2019), while other researchers examine how the design and features of context could allow competition and collaboration between members to generate quality creative ideas online (Martinez, 2017; Renard and Davis, 2019).

#### A technological perspective

A technological perspective that examines creative ideas generation within VCs focuses on technical issues, such as the information technologies being adopted (Yuan et al., 2016; Chen et al., 2021). Studies adopting this perspective often aim to enhance the user experience and quality of service offered by the online community or platform. Consistent with previous studies (Füller et al., 2019; Priharsari and Abedin, 2021; Ataman and Tuncer, 2022), we divide technological factors into instrumental and non-instrumental qualities. Instrumental qualities concern the perceived support that the tool, platform, or system provides to help users accomplish their tasks and goals, mainly including the perceived ease of use/network size, privacy risk/control, functional value, and technological interactivity. Non-instrumental qualities of the system mainly involve some factors related to human-computer interaction, such as social interactivity, media sociability, and perceived entertainment/aesthetic appeal (Füller et al., 2019). Our data suggests that research adopting a technological perspective of online creative ideas generation tends to focus on the two aforementioned subthemes: instrumental and non-instrumental qualities. For example, some studies look at how functions or features of social media platforms can promote creative ideas generation among virtual members (Foroudi et al., 2019; Zhang et al., 2019), while others examine the perceived entertainment level of virtual members as they engage with the social media platforms and how these platforms can impact their creative ideas generation process (Hussain et al., 2022).

### Which guiding theories have been adopted by researchers in relation to the three perspectives?

**Figure 7:**
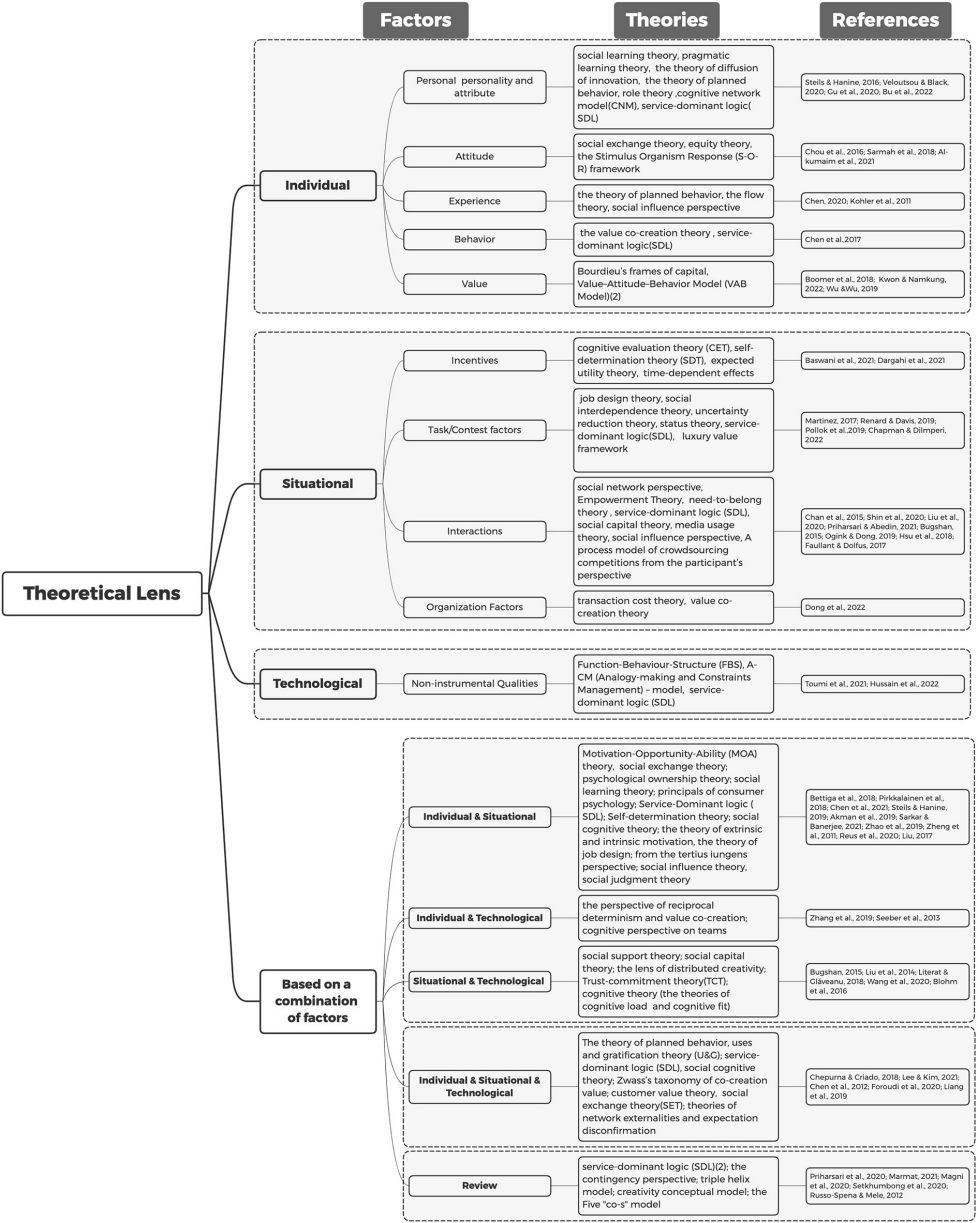
Selected articles by theoretical lens and influencing factors.

Given the importance of theories, we also analyzed the theories upon which the selected studies formulated their propositions and hypotheses regarding the three identified perspectives. Figure 7 presents an overview of theories as well as a list of articles that adopted these theories. The theories involved were analyzed from individual, situational, and technological perspectives and a combination of two perspectives and all three perspectives. In addition, theories mentioned in reviews of relevant topics were included.

As can be seen from Figure 7, service-dominant logic (SDL) is the most widely used theory (Akman et al., 2019; Baswani et al., 2021; Bu et al., 2022) across all perspectives. According to SDL theory, value is co-created by the interaction of multiple producers and consumers through the integration of resources and their respective capabilities (Vargo and Lusch, 2008). As creative ideas generation increasingly focuses on the consumer's experience (Prahalad and Ramaswamy, 2004), research which is based on SDL becomes more extensive.

For studies approached solely from an individual perspective, the theories involved mainly focus on attitude, experience, behavior, value, and individual personality/attributes. Planned behavior theory (*n* = 2) and the Value-Attitude-Behavior (VAB) Model (*n* = 2) are widely used. For studies approached solely from a situational perspective, the theories involved are mainly related to motivation, interaction, and organizational factors. SDL is the most widely used theory (*n* = 4), followed by value co-creation theory (*n* = 2). For studies focusing only on the technological perspective, relevant theories are included under non-instrumental qualities factors and mainly adopt the SDL, Function-Behavior-Structure, and ACM models.

There are 10 studies that approach their respective research from the individual-situation perspective, which is mainly based on the motivation theory [e.g., Motivation-Opportunity-Ability (MOA) theory, the theory of extrinsic and intrinsic motivation] and cognitive theory (e.g., social learning theory, social cognitive theory). Meanwhile, research using the individual-technological perspective is mainly based on the reciprocal and cognitive theories. Cognitive theory is primarily used in research that uses the situational-technological perspective. Five studies simultaneously incorporated the individual, situational, and technological perspectives. The theories involved included the planned behavior, SDL, social cognitive, and customer value theories. In addition, we also carried out an analysis of the selected reviews, which showed that two studies used SDL as a theoretical framework and included the triple helix model, the creativity conceptual model, and the five “co-s” model. Unfortunately, 8 of the 68 articles used a theoretical lens that is still unclear.

## Discussion

This study analyses and discusses creative ideas generation in VCs through a systematic literature review, while seeking to gain a clearer picture of the current state of research on the topic by providing an overview of the factors that influence the creative ideas generation in VCs. Research trends, driving factors, and associated main theories and methods have been identified through the systematic review. The results of this review could be used to guide organizations and individuals with a role or interest in creative ideas generation within VCs on how to efficiently and effectively gather ideas. This review also provides research trends and gaps in the literature for academics who are interested in conducting future research in this area.

Our research results suggest there has been a steady increase in the number of articles published on this topic, especially from scholars based in China, the US, and the UK. This could be arguably due to the increased popularity of social media platforms within these regions (Bugshan, 2015). Although most of these studies have been carried out in commercial settings (e.g., online crowdsourcing), research attention has been growing in non-commercial settings (e.g., education, health, government), especially over the past 5 years. This is consistent with the literature, which suggests that there is an increase in the number of VCs associated with the advancement of information technology (Varshney, 2014) in the fields of health (Liu et al., 2020; Mirzaei and Esmaeilzadeh, 2021), entertainment (Chou et al., 2016; Chen, 2020), and education (Al-kumaim et al., 2021). Therefore, future research on creative ideas generation in online communities in non-commercial contexts can be strengthened.

Similar to most studies conducted in commercial settings, we discovered that transaction-induced VCs received the most research attention. These studies mainly concentrated on crowdsourcing or contests, firm-sponsored communities, and product open innovation communities (Faullant and Dolfus, 2017; Baswani et al., 2021). Through online interactions with consumers, companies can enhance their brand reputations, develop new business ideas, achieve successful innovations, and enlarge their customer pools (Fisher, 2019; Park et al., 2019). There is a relatively large amount of research on interest and fantasy-based VCs. However, there is also a lack of research on relationship-based VCs. These VCs offer opportunities for people to share experiences and form meaningful personal relationships (e.g., online health communities) (Goh et al., 2016). This pattern is becoming more prevalent in the current context, especially as many patients and their families cannot go to hospitals due to the COVID-19 pandemic. Future studies should explore this context in more detail.

In terms of research design, most studies adopted quantitative methods, although some qualitative studies were conducted. Despite that the mixed-methods approach can enhance the reliability of results and the robustness of data (Hutter et al., 2011), there is still a lack of mixed-methods research being conducted, especially in commercial settings or transaction-induced VCs. Future research could build on these gaps and contribute to our understanding of online creative ideas generation in these areas.

Our results also identified three major research perspectives when studying online creative ideas generation, namely: individual, situational, and technological perspectives. Unfortunately, most studies adopt a single perspective rather than combining multiple perspectives. For example, studies from a situational perspective tend to focus on contributing factors to online creative ideas generation, such as incentives, organizational factors, environmental factors, etc., while overlooking other perspectives such as individual and technological. With the rise of interactive digital technology media, users and consumers rely on computer-mediated technology to generate creative ideas in online communities (Lee and Kim, 2022). However, research that examines multiple perspectives (e.g., individual-technological, situational-technological, or a combination of three perspectives) is still limited. More studies should adopt multiple perspectives when investigating online creative ideas generation in the future.

Regarding the theoretical foundation, our study suggests that many researchers drew on theories from diverse research disciplines. This could be arguable to the vast number of contributing factors that have been identified. Consistent with previous studies (Priharsari and Abedin, 2021), we found that service-dominant logic (SDL) was the most widely used theory among our selected studies. In addition, cognitive theory, planned behavior theory, and value-related theory are also frequently used for studying online creative ideas generation.

The findings of this systematic literature review should be read in light of some research limitations. First, our search only incorporated studies published in English. This might exclude studies that have been published in other languages. Secondly, our review only focused on peer-reviewed journal articles to ensure the rigor of research findings. However, this may exclude important theoretical work communicated through books or high-quality conference proceedings. Finally, the scope of this review is mainly conducted through a quantitative approach to provide an overview of studies conducted on the topic. Future research could build on this work and explore the lens of narrative data.

## Data availability statement

The original contributions presented in the study are included in the article/supplementary material, further inquiries can be directed to the corresponding author.

## Author contributions

XZ and CW conducted literature review, data collection and analysis, wrote, and revised the manuscript. JH conceptualized and gave research aim, questions, and recommendations. All authors contributed to the article and approved the submitted version.

## Conflict of interest

The authors declare that the research was conducted in the absence of any commercial or financial relationships that could be construed as a potential conflict of interest.

## Publisher's note

All claims expressed in this article are solely those of the authors and do not necessarily represent those of their affiliated organizations, or those of the publisher, the editors and the reviewers. Any product that may be evaluated in this article, or claim that may be made by its manufacturer, is not guaranteed or endorsed by the publisher.

## References

[B1] Akman H. Plewa C. Conduit J. (2019). Co-creating value in online innovation communities. EJM 53, 1205–1233. 10.1108/EJM-12-2016-0780

[B2] Al-kumaim N. H. Alhazmi A. K. Ramayah T. Shabbir M. S. Gazem N. A. (2021). Sustaining continuous engagement in value co-creation among individuals in universities using online platforms: role of knowledge self-efficacy, commitment and perceived benefits. Front. Psychol. 12, 637808. 10.3389/fpsyg.2021.637808 33643168 PMC7907507

[B3] Ataman C. Tuncer B. (2022). Urban interventions and participation tools in urban design processes: a systematic review and thematic analysis (1995 – 2021). Sustain. Cities Soc. 76, 103462. 10.1016/j.scs.2021.103462

[B4] Baer M. (2012). Putting creativity to work: the implementation of creative ideas in organisations. AMJ 55, 1102–1119. 10.5465/amj.2009.0470

[B5] Barrett M. S. Creech A. Zhukov K. (2021). Creative collaboration and collaborative creativity: a systematic literature review. Front. Psychol. 12, 713445. 10.3389/fpsyg.2021.713445 34434151 PMC8380918

[B6] Baswani S. Townsend A. M. Luse A. (2021). Company-sponsored online co-creation and financial incentives: the impact of intrinsic motivation on participation intention. Int. J. Electron. Commerce 25, 394–415. 10.1080/10864415.2021.1967002

[B7] Bettiga D. Lamberti L. Noci G. (2018). Investigating social motivations, opportunity and ability to participate in communities of virtual co-creation. Int. J. Consum. Stud. 42, 155–163. 10.1111/ijcs.12409

[B8] Bu Y. Parkinson J. Thaichon P. (2022). Influencer marketing: homophily, customer value co-creation behaviour and purchase intention. J. Retail. Consumer Serv. 66, 102904. 10.1016/j.jretconser.2021.102904

[B9] Bugshan H. (2015). Co-innovation: the role of online communities. J. Strateg. Mark. 23, 175–186. 10.1080/0965254X.2014.92090528502399

[B10] Calic G. Shevchenko A. Ghasemaghaei M. Bontis N. Tokcan Z. O. (2020). From sustainability constraints to innovation: enhancing innovation by simultaneously attending to sustainability and commercial imperatives. Sustain. Account. Manage. Pol. J. 11, 695–715. 10.1108/SAMPJ-02-2019-0084

[B11] Chen A. Jin Y. Xiang M. Lu Y. (2021). Online value co-creation activities in three management domains: the role of climate and personal needs. Int. J. Consum. Stud. 46, 1339–1364. 10.1111/ijcs.12761

[B12] Chen C. Du R. Li J. Fan W. (2017). The impacts of knowledge sharing-based value co-creation on user continuance in online communities. Inf. Discov. Deliv. 45, 227–239. 10.1108/IDD-11-2016-0043

[B13] Chen H. Hu Y. J. Huang S. (2019). Monetary incentive and stock opinions on social media. J. Manage. Inform. Syst. 36, 391–417. 10.1080/07421222.2019.1598686

[B14] Chen Y. W. (2020). Sustainable value co-creation in the virtual community: How diversified co-creation experience affects co-creation intention. Int. J. Environ. Res. Public Health 17, 8497. 10.3390/ijerph17228497 33212782 PMC7696490

[B15] Chou E. Y. Lin C. Y. Huang H. C. (2016). Fairness and devotion go far: Integrating online justice and value co-creation in virtual communities. Int. J. Inf. Manag. 36, 60–72. 10.1016/j.ijinfomgt.2015.09.009

[B16] Faullant R. Dolfus G. (2017). Everything community? Destructive processes in communities of crowdsourcing competitions. BPMJ 23, 1108–1128. 10.1108/BPMJ-10-2016-0206

[B17] Fisher G. (2019). Online communities and firm advantages. Acad. Manage. Rev. 44, 279–298. 10.5465/amr.2015.029028139283

[B18] Foroudi P. Cuomo M. T. Foroudi M. M. (2019). Continuance interaction intention in retailing: relations between customer values, satisfaction, loyalty, and identification. ITP 33, 1303–1326. 10.1108/ITP-09-2018-0421

[B19] Füller K. Weking J. Böhm M. Krcmar H. (2019). Leveraging customer-integration experience: a review of influencing factors and implications. CAIS 44, 81–128. 10.17705/1CAIS.04404

[B20] Girotra K. Terwiesch C. Ulrich K. T. (2010). Ideas generation and the quality of the best idea. Manage. Sci. 56, 591–605. 10.1287/mnsc.1090.1144

[B21] Goh J. M. Gao G. Agarwal R. (2016). The creation of social value: Can an online health community reduce rural-urban health disparities? MISQ 40, 247–263. 10.25300/MISQ/2016/40.1.11

[B22] Gu C. Han M. Li C. Bie Z. Tan Y. Xue Y. . (2020). The effect of environmental cues and motivation on creative ideas generation. Creat. Innov. Manage. 29, 581–596. 10.1111/caim.12403

[B23] Hagel J. Armstrong A. (1997). Net Gain: Expanding Markets Through Virtual Communities. Boston, MA: Harvard Business Press.

[B24] Han J. Park D. Forbes H. Schaefer D. (2020). A computational approach for using social networking platforms to support creative ideas generation. Proc. CIRP 91, 382–387. 10.1016/j.procir.2020.02.190

[B25] Hawlina H. Gillespie A. Zittoun T. (2019). Difficult differences: a socio-cultural analysis of how diversity can enable and inhibit creativity. J. Creat. Behav. 53, 133–144. 10.1002/jocb.182

[B26] Hussain A. Ting D. H. Mazhar M. (2022). Driving consumer value co-creation and purchase intention by social media advertising value. Front. Psychol. 13, 800206. 10.3389/fpsyg.2022.800206 35282229 PMC8912946

[B27] Hutter K. Hautz J. Füller J. Mueller J. Matzler K. (2011). Communitition: The tension between competition and collaboration in community-based design contests. Creat. Innov. Manage. 20, 3–21. 10.1111/j.1467-8691.2011.00589.x

[B28] Kay G. (1995). Effective meetings through electronic brainstorming. J. Manage. 35, 4–25. 10.1108/02621719510086147

[B29] Lee J. Ahn J. Kim J. Kho J. (2019). Co-design education based on the changing designer's role and changing creativity. Int. J. Art. Des. Educ. 38, 430–444. 10.1111/jade.12204

[B30] Lee Y. Kim D. (2022). The influence of technological interactivity and media sociability on sport consumer value co-creation behaviors via collective efficacy and collective intelligence. IJSMS 23, 18–40. 10.1108/IJSMS-04-2020-0058

[B31] Liao J. Wang H.-C. (2019). Gestures as intrinsic creativity support: understanding the usage and function of hand gestures in computer-mediated group brainstorming. Proc. ACM Hum. Comput. Interact. 3, 1–16. 10.1145/336112434322658

[B32] Liu S. Xiao W. Fang C. Zhang X. Lin J. (2020). Social support, belongingness, and value co-creation behaviors in online health communities. Telemat. Inform. 50, 101398. 10.1016/j.tele.2020.101398

[B33] Marmat G. (2021). Enhancing brand experience in the online social media network context: a contingency perspective. QMR 24, 581–609. 10.1108/QMR-07-2020-0096

[B34] Martinez M. G. (2017). Inspiring crowdsourcing communities to create novel solutions: competition design and the mediating role of trust. Technol. Forecast. Soc. Change 117, 296–304. 10.1016/j.techfore.2016.11.015

[B35] Martínez-López F. J. Anaya-Sánchez R. Aguilar-Illescas R. Molinillo S. (2016). “Types of virtual communities and virtual brand communities,” in Online Brand Communities, ed B. Bethke (Cham: Springer), 125–140.

[B36] McGrath L. Bresciani S. Eppler M. J. (2016). We walk the line: icons provisional appearances on virtual whiteboards trigger elaborative dialogue and creativity. Comput. Hum. Behav. 63, 717–726. 10.1016/j.chb.2016.05.086

[B37] Mirzaei T. Esmaeilzadeh P. (2021). Engagement in online health communities: Channel expansion and social exchanges. Inform. Manage-Amster. 58, 103404. 10.1016/j.im.2020.103404

[B38] Mitchell I. K. Walinga J. (2017). The creative imperative: the role of creativity, creative problem solving and insight as key drivers for sustainability. J. Clean. Prod. 140, 1872–1884. 10.1016/j.jclepro.2016.09.162

[B39] Mumford M. D. Mobley M. I. Reiter-Palmon R. Uhlman C. E. Doares L. M. (1991). Process analytic models of creative capacities. Creat. Res. J. 4, 91–122. 10.1080/10400419109534380

[B40] Nevo S. Nevo D. Pinsonneault A. (2020). Exploring the role of IT in the front-end of innovation: an empirical study of it-enabled creative behavior. Inform. Org. 30, 100322. 10.1016/j.infoandorg.2020.100322

[B41] Oldham G. R. Da Silva N. (2015). The impact of digital technology on the generation and implementation of creative ideas in the workplace. Comput. Human Behav. 42, 5–11. 10.1016/j.chb.2013.10.041

[B42] Osborn A. F. (1957). Applied Imagination, 1st Edn. New York: Scribner.

[B43] Page M. J. McKenzie J. E. Bossuyt P. M. Boutron I. Hoffmann T. C. Mulrow C. D. . (2021). The PRISMA 2020 statement: an updated guideline for reporting systematic reviews. Int. J. Surg. 88, 105906. 10.1016/j.ijsu.2021.105906 33789826

[B44] Park E. Im G. Storey V. C. Baskerville R. L. (2019). Never, never together again: How postpurchase affect drives consumer outcomes within the context of online consumer support communities. J. Assoc. Inf. Syst. 20, 58–104. 10.17705/1jais.00529

[B45] Paulus P. B. Yang H.-C. (2000). Ideas generation in groups: a basis for creativity in organisations. Org. Behav. Hum. Decision Processes 82, 76–87. 10.1006/obhd.2000.2888

[B46] Piyathasanan B. Mathies C. Patterson P. G. de Ruyter K. (2018). Continued value creation in crowdsourcing from creative process engagement. J. Serv. Mark. 32, 19–33. 10.1108/JSM-02-2017-0044

[B47] Porter C. E. (2004). A typology of virtual communities: a multi-disciplinary foundation for future research. JCMC 10, JCMC1011. 10.1111/j.1083-6101.2004.tb00228.x

[B48] Prahalad C. K. Ramaswamy V. (2004). Co-creation experiences: the next practice in value creation. J. Interact. Mark. 18, 5–14. 10.1002/dir.20015

[B49] Priharsari D. Abedin B. (2021). Orchestrating value co-creation in online communities as fluid organisations: firm roles and value creation mechanisms. ITP. 10.1108/ITP-10-2020-0707. [Epub ahead of print].

[B50] Rashid Y. Waseem A. Akbar A. A. Azam F. (2019). Value co-creation and social media: a systematic literature review using citation and thematic analysis. EBR 31, 761–784. 10.1108/EBR-05-2018-0106

[B51] Renard D. Davis J. G. (2019). Social interdependence on crowdsourcing platforms. J. Bus. Res. 103, 186–194. 10.1016/j.jbusres.2019.06.033

[B52] Richard P. Burkhardt J. M. Lubart T. Bourgeois-Bougrine S. Barr,é J. (2018). “The effect of virtual environment and user/designer collaboration on the creative co-design process,” in Congress of the International Ergonomics Association (Cham. Springer), 605–614.

[B53] Ridings C. M. Gefen D. Arinze B. (2002). Some antecedents and effects of trust in virtual communities. J. Strategic Inform. Syst. 11, 271–295. 10.1016/S0963-8687(02)00021-5

[B54] Schuhmacher M. C. Kuester S. (2012). Identification of lead user characteristics driving the quality of service innovation ideas: identification of lead user characteristics. Creat. Innov. Manag. 21, 427–442. 10.1111/caim.12002

[B55] Seeber I. Maier R. Weber B. (2013). Macrocognition in collaboration: analysing processes of team knowledge building with CoPrA. Group Decis. Negot. 22, 915–942. 10.1007/s10726-012-9337-z

[B56] Shan W. Qiao T. Zhang M. (2020). Getting more resources for better performance: the effect of user-owned resources on the value of user-generated content. Technol. Forecast. Soc. Change 161, 120318. 10.1016/j.techfore.2020.120318

[B57] Škerlavaj M. Cerne M. Dysvik A. (2014). I get by with a little help from my supervisor: creative-ideas generation, idea implementation, and perceived supervisor support. Leadership Q. 25, 987–1000. 10.1016/j.leaqua.2014.05.003

[B58] Somech A. Drach-Zahavy A. (2013). Translating team creativity to innovation implementation: the role of team composition and climate for innovation. J. Manag. 39, 684–708. 10.1177/0149206310394187

[B59] Vargo S. L. Lusch R. F. (2008). Service-dominant logic: continuing the evolution. J. Acad. Mark. Sci. 36, 1–10. 10.1007/s11747-007-0069-6

[B60] Varshney U. (2014). Mobile health: four emerging themes of research. Decis. Support. Syst. 66, 20–35. 10.1016/jdss.2014.06.001

[B61] Yuan D. Lin Z. Zhuo R. (2016). What drives consumer knowledge sharing in online travel communities?: personal attributes or e-service factors? Comput. Hum. Behav. 63, 68–74. 10.1016/j.chb.2016.05.019

[B62] Zhang W. Zhang Q. Song M. (2015). How do individual-level factors affect the creative solution formation process of teams?: creative solution formation process of teams. Creat. Innov. Manag. 24, 508–524. 10.1111/caim.12127

[B63] Zhang Y. Zhang M. Luo N. Wang Y. Niu T. (2019). Understanding the formation mechanism of high-quality knowledge in social question and answer communities: a knowledge co-creation perspective. Int. J. Inf. Manag. 48, 72–84. 10.1016/j.ijinfomgt.2019.01.022

[B64] Zheng H. Li D. Hou W. (2011). Task design, motivation, and participation in crowdsourcing contests. Int. J. Electro. Commerce 15, 57–88. 10.2753/JEC1086-4415150402

[B65] Zhu H. Kock A. Wentker M. Leker J. (2019). How does online interaction affect idea quality? The effect of feedback in firm-internal idea competitions: the effect of feedback in firm-internal idea competitions. J. Prod. Innov. Manag. 36, 24–40. 10.1111/jpim.12442

